# Supplementary material for: Simultaneous Silencing of Gut Nucleases and a Vital Target Gene by Adult dsRNA Feeding Enhances RNAi Efficiency and Mortality in Ceratitis capitata
Source: Insects. 2024 Sep 19;15(9):717. doi: 10.3390/insects15090717 (PMC11432467; doi:10.3390/insects15090717)
Supplement: Supplementary file 1 [file insects-15-00717-s001.zip › insects-3163290-supplementary.pdf]

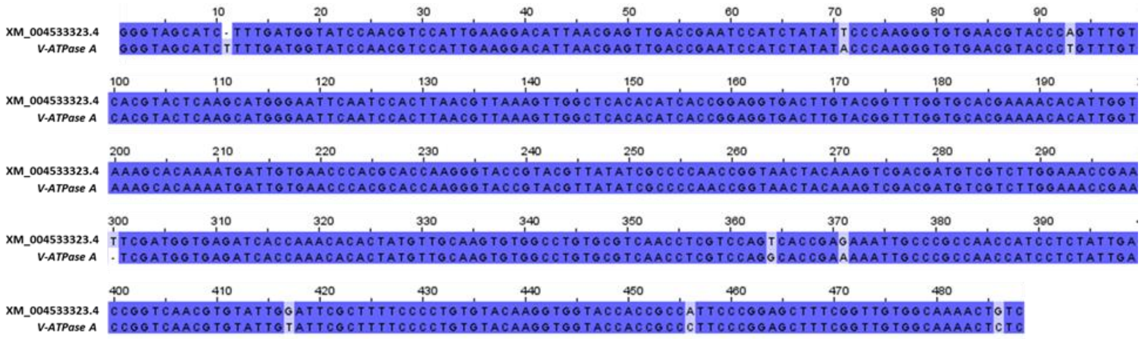

Figure S1. Alignment between dsRNA sequence of *v-ATPase A (CcVha68-1)* gene and XM_004533323.4 (NCBI).

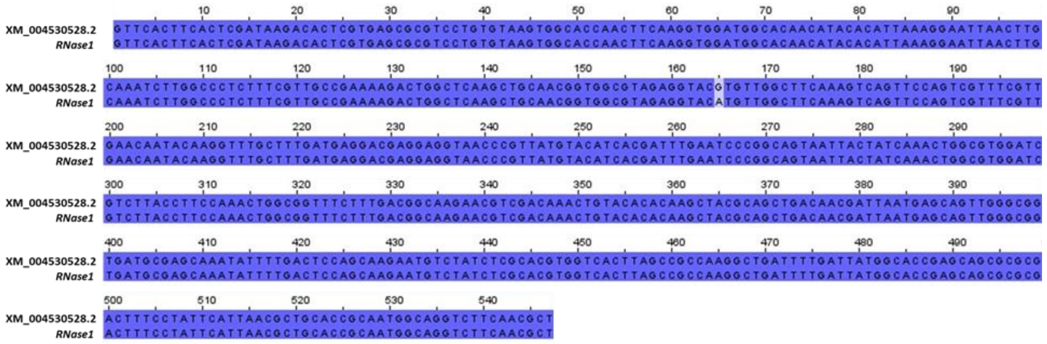

Figure S2. Alignment between dsRNA sequence of *RNase1 (CcdsRNase1)* gene and XM_004530528.2 (NCBI).

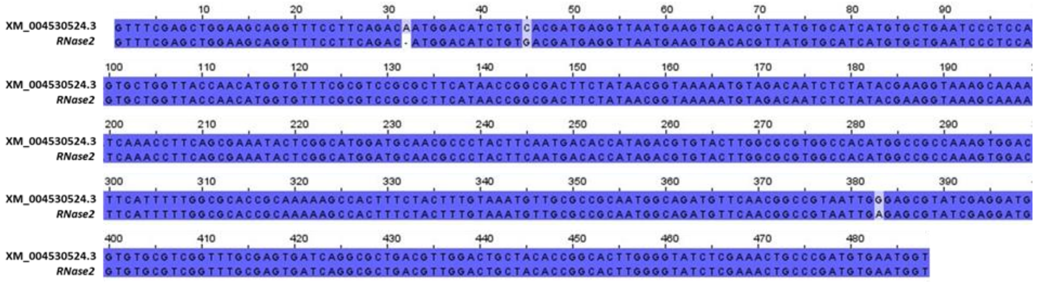

Figure S3. Alignment between dsRNA sequence of *RNase2 (CcdsRNase2)* gene and XM_004530524.3 (NCBI).

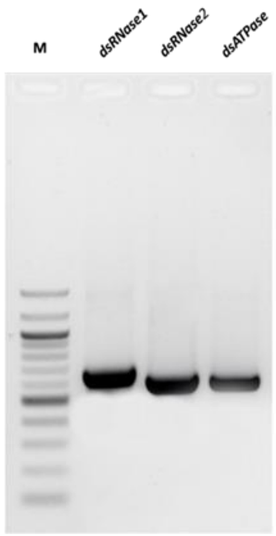

Figure S4. Agarose gel of the synthesized dsRNAs. Lane 1: marker 100 bp; lane 2: dsRNase1(613 bp); lane 3: dsRNase2 (557 bp); lane 4: dsATPase (553 bp).

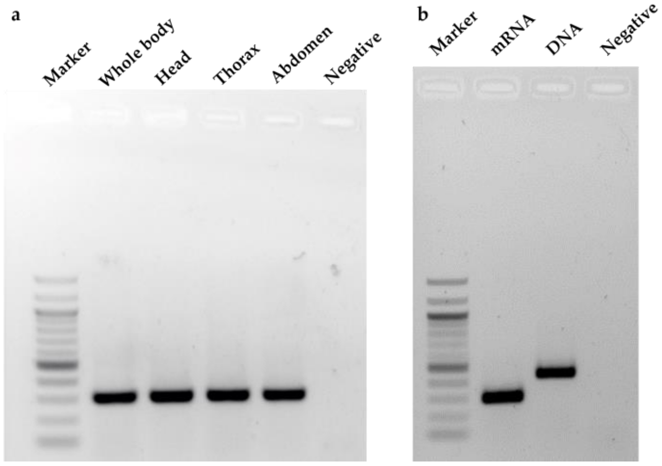

Figure S5. Agarose gel of *CcSOD* gene (housekeeping) expression in different adult tissues. (A) shows a 300 bp band in all samples; lane 1: marker 100 bp; lane 2: whole body; lane 3: head; lane 4: thorax; lane 5: abdomen; lane 6: negative control; (B) shows the difference between the presence/absence of an intron-sequence; lane 1: marker 100 bp; lane 2: mRNA (cDNA); lane 3: DNA (genomic); lane 4: negative control.

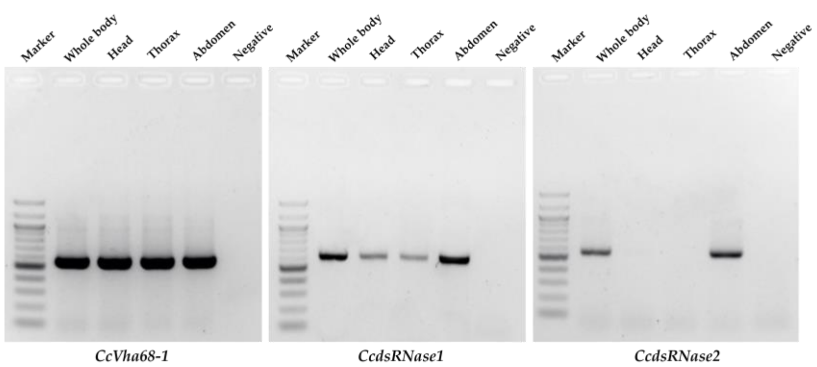

Figure S6. Agarose gel of *CcVha68-1*, *CcdsRNase1*, and *CcdsRNase2* gene expression in different adult tissues. For each gel: lane 1: marker 100 bp; lane 2: whole body; lane 3: head; lane 4: thorax; lane 5: abdomen; lane 6: negative control.

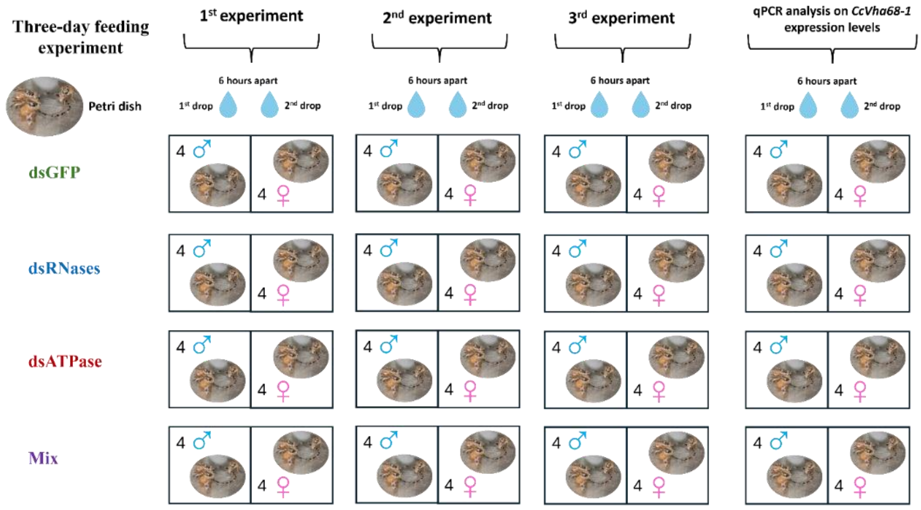

Figure S7. Schematic illustration of the mortality assay experiment.

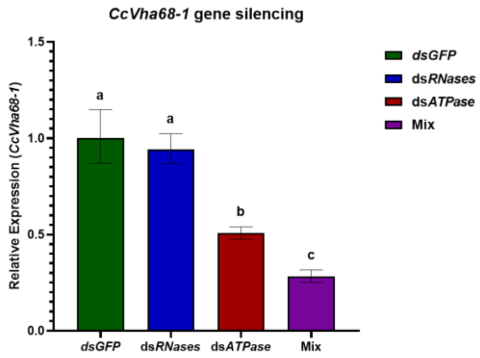

Figure S8. Transcript levels of *CcVha68-1* gene after three-day feeding experiment (one-way ANOVA: $F(3,24) = 34.84$, $P < 0.0001$). Different letters denote a significant difference between mean values recorded for each group (the obtained values passed normality tests). The values reported are the mean ± standard error.

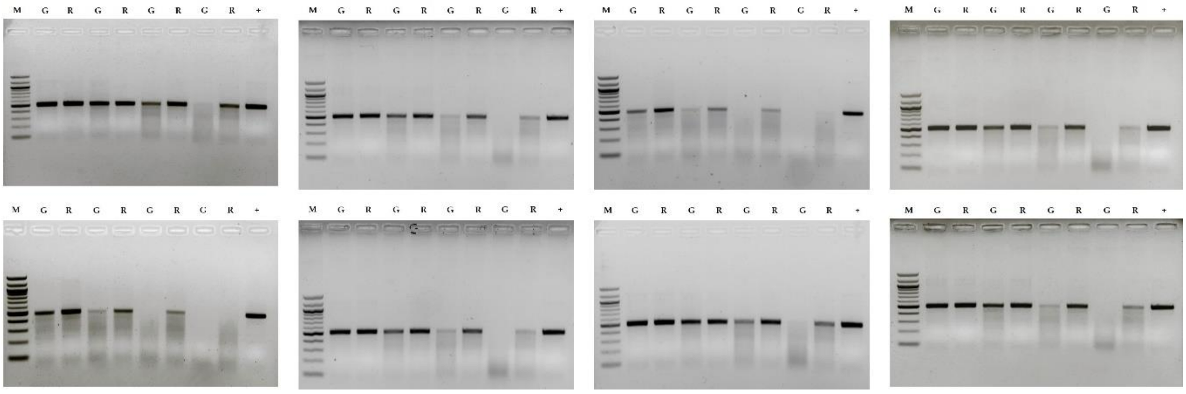

Figure S9. Agarose gels of *ex vivo* experiment. M: marker; G: dsGFP; R: dsRNases; +: positive control (dsATPase, 100 ng).

Primers	Sequences	Amplicon size (bp)	Tm (°C)
**T7_dsRNase1_Fwd**	TAATACGACTCACTATAGGGAGA GGATTTGTGGTGTCCCGGTA	613 bp	60
**T7_dsRNase1_Rev**	TAATACGACTCACTATAGGGAGA GCGTTGAAGACCTGCCATTG		
**T7_dsRNase2_Fwd**	TAATACGACTCACTATAGGGAGA CGGCACTGATCTTCTGGAGG	557 bp	60
**T7_dsRNase2_Rev**	TAATACGACTCACTATAGGGAGA ACCATTCACATCGGGCAGTT		
**T7_dsATPase_Fwd**	TAATACGACTCACTATAGGGAGA TGTTGAATTGGGTCCCGGTA	553 bp	60
**T7_dsATPase_Rev**	TAATACGACTCACTATAGGGAGA GTTTTGCCACAACCGAAAGC		
**CcSOD_Fwd**	TGCTCCGAGAACGTTCACG	300 bp	60
**CcSOD_Rev**	TCATCGGTCAATTCGTGCAC		

Table S1. Primers for *CcSOD* gene amplification and the synthesis of dsRNAs target.

Primers	Sequences	Amplicon size (bp)	Tm (°C)
**RT_RNase1_Fwd**	ATTCATTAACGCTGCACCGC	118 bp	60
**RT_RNase1_Rev**	ACCCGTCCAGCAGTCTACAT		
**RT_RNase2_Fwd**	GGTCAAATATTCCGTGCCGC	113 bp	60
**RT_RNase2_Rev**	TAGCTGGTGTACTCGGTCCA		
**RT_ATPase_Fwd**	TCCCCGAACTTACTTGCGAA	83 bp	60
**RT_ATPase_Rev**	CATGTTGGAGGTGTTGGCAA		
**Rpl19_FWD**	AACAAACGTTGTACTGATGG	103 bp	60
**Rpl19_REV**	CACGTACTTTATGTCGTCTG		

Table S2. Primers for qPCR analysis.

dsGFP	dsRNases	dsATPase	Mix
ddH$_2$O	ddH$_2$O	ddH$_2$O	ddH$_2$O
Sucrose 10%	Sucrose 10%	Sucrose 10%	Sucrose 10%
dsGFP (200 ng/μL)	dsRNase1 (100 ng/μL)	dsATPase (200 ng/μL)	dsRNase1 (100 ng/μL)
/	dsRNase2 (100 ng/μL)	/	dsRNase2 (100 ng/μL)
/	/	/	dsATPase (200 ng/μL)

Table S3. dsRNAs feeding experimental groups.

Additional information: aminoacidic sequences for phylogenetic analyses

Vha68-1 aa sequences

>Dmel_NP_001260426.1

MSNLRKFKDEERESEYGRVYAVSGPVVTAEAMSGSAMYELVRVGYYELVGEIIRLEGDMATIQVYEETSGVTVGDPVLRTGK
PLSVELGPGIMGSIFDGIQRPLRDIGVMTNSIYIPKGVNTTALSRSEMWEFNPLNVRVGSHITGGDLYGVVHENTLVKQRMI
VAPRAKGTVRYIAPAGNYNLEDIVLETEFDGEITKHTMLQVWPVRQPRPVTEKLPANHPLFTGQRVLDSLFPCVQGGTTAIP
GAFGCGKTVISQALSKYSNSDVIIYVGCGERGNEMSEVLRDFPELTCEIDGVTESIMKRTALVANTSNMPVAAREASIYTGITL
SEYFRDMGYNVAMMADSTSRWAEALREISGRLAEMPADSGYPAYLGARLATFYERAGRVKCLGNPEREGSVSIVGAVSPPG
GDFSDPVTSATLGIVQVFWGLDKKLAQRKHFPSINWLISYSKYMRALDEYYDKNYPEFVPLRTKVKEILQEEEDLSEIVQLVGK
ASLAETDKVTLEVAKLLKDDFLQQNSYSPYDRVCPFYKTVGMLRNIMAFYETARHAVESTAQSDNKITWNTIRESMGGIMY
QLSSMKFKDPVKDGEQKIKADYDQLYEDLQQAFRNLED

>Ccap_XP_004533376.1

MSTLKCYQDEERESQYGRVYAVSGPVVTAERMSGSAMYELVRVGYYELVGEIIRLEGDMATIQVYEETSGVTVGDPVLRTGK
PLSVELGPGILGSIFDGIQRPLKDIGESSGSIYIPKGVNIPSLSRTEAWEFNPTNVRVGSHITGGDLYGIVQENTLVKHRLIVGPR
CKGTVRYIAPPGNYTVEDVILETEFDGEITKHTMLQVWPVRQPRPVTEKLPANHPLFTGQRVLDSLFPCVQGGTTAIPGAFG
CGKTVISQALSKYSNSDVIIYVGCGERGNEMSEVLRDFPELTCEIDGVVESIMKRTALVANTSNMPVAAREASIYTGITLSEYFR
DMGYNVAMMADSTSRWAEALREISGRLAEMPADSGYPAYLGARLASFYERAGRVKCLGNPEREGSVSIVGAVSPPGGDFS
DPVTSATLGIVQVFWGLDKKLAQRKHFPSINWLISYSKYMRALDEYYDKNFPEFVPLRTKVKEILQEEEDLSEIVQLVGKASLA
ETDKITLEVAKLVKDDFLQQNSYSPYDRVCPFYKTVGMLRNMLAFYELARHAVESTAQSDNKITWNTIRDAMGNILYQLSSM
KFKDPVKDGEAKIKADYEQLHEDLQQAFRNLED

>Btry_XP_039950063.1

MSTLKCYQDEERESQYGRVFAVSGPVVTAERMSGSAMYELVRVGYYELVGEIIRLEGDMATIQVYEETSGVTVGDPVLRTGK
PLSVELGPGILGSIFDGIQRPLKDIGESSGSIYIPKGVNIPSLSRTEAWDFNPTNVRVGSHITGGDLYGIVHENTLVKHRLIVGPR
CKGTVRYIAPAGNYTVEDVILETEFDGEITKHTMLQVWPVRQPRPVTEKLPANHPLFTGQRVLDSLFPCVQGGTTAIPGAFG
CGKTVISQALSKYSNSDVIIYVGCGERGNEMSEVLRDFPELTCEIDGVVESIMKRTALVANTSNMPVAAREASIYTGITLSEYFR
DMGYNVAMMADSTSRWAEALREISGRLAEMPADSGYPAYLGARLASFYERAGRVKCLGNPEREGSVSIVGAVSPPGGDFS
DPVTSATLGIVQVFWGLDKKLAQRKHFPSINWLISYSKYMRALDEYYDKNFPEFVPLRTKVKEILQEEEDLSEIVQLVGKASLA

ETDKITLEVAKLVKDDFLQQNSYSPYDRVCPFYKTVGMLRNMLAFYELARHAVESTAQSDNKITWNVIRDAMGNILYQLSS
MKFKDPVKDGEAKIKADYEQLHEDLQQAFRNLED

>Zcuc_XP_011178667.1

MSTLKCYQDEERESQYGRVYAVSGPVVTAERMSGSAMYELVRVGYYELVGEIIRLEGDMATIQVYEETSGVTVGDPVLRTGK
PLSVELGPGILGSIFDGIQRPLKDIGESSGSIYIPKGVNIPALSRTEAWDFNPTNVRVGSHITGGDLYGIVHENTLVKHRLIVGPR
CKGTVRYIAPAGNYTVEDVILETEFDGEITKHTMLQVWPVRQPRPVTEKLPANHPLFTGQRVLDSLFPCVQGGTTAIPGAFG
CGKTVISQALSKYSNSDVIIYVGCGERGNEMSEVLRDFPELTCEIDGVVESIMKRTALVANTSNMPVAAREASIYTGITLSEYFR
DMGYNVAMMADSTSRWAEALREISGRLAEMPADSGYPAYLGARLASFYERAGRVKCLGNPEREGSVSIVGAVSPPGGDFS
DPVTSATLGIVQVFWGLDKKLAQRKHFPSINWLISYSKYMRALDEYYDKNFPEFVPLRTKVKEILQEEEDLSEIVQLVGKASLA
ETDKITLEVAKLVKDDFLQQNSYSPYDRVCPFYKTVGMLRNMLAFYELARHAVESTAQSDNKITWNVIRDAMGNILYQLSS
MKFKDPVKDGEAKIKADYEQLHEDLQQAFRNLED

>Alud_XP_053958742.1

MSTLKCYQDEERESQYGRVYAVSGPVVTAERMSGSAMYELVRVGYYELVGEIIRLEGDMATIQVYEETSGVTVGDPVLRTGK
PLSVELGPGILGSIFDGIQRPLKDIGESSGSIYIPKGVNIPALSRTQAWEFNPTNVRVGSHITGGDLYGLVHENTLVKHRLIVGPR
CKGTVRYMAPAGNYTVEDVILETEFDGEITKHTMLQVWPVRQPRPCTEKLPANHPLFTGQRVLDSLFPCVQGGTTAIPGAF
GCGKTVISQALSKYSNSDVIIYVGCGERGNEMSEVLRDFPELTCEIDGVIESIMKRTALVANTSNMPVAAREASIYTGITLSEYF
RDMGYNVAMMADSTSRWAEALREISGRLAEMPADSGYPAYLGARLASFYERAGRVKCLGNPEREGSVSIVGAVSPPGGDF
SDPVTSATLGIVQVFWGLDKKLAQRKHFPSINWLISYSKYMRALDEYYDKNFPEFVPLRTKVKEILQEEEDLSEIVQLVGKASL
AETDKITLEVAKLVKDDFLQQNSYSPYDRVCPFYKTVGMLRNMLAFYELARHAVESTAQSDNKITWNVIRDAMGNILYQLSS
MKFKDPVKDGEAKIKADYEQLHEDLQQAFRNLED

>Ldec_XP_023012283.1

MSRLIADEEQESQYGYVHAVSGPVVTAEKMSGSAMYELVRVGYYELVGEIIRLEGDMATIQVYEETSGVTVGDPVLRTGKPL
SVELGPGIMGSIFDGIQRPLKDICDMTESIYIPKGVNVPSLSRTIKWEFNPINIKLGSHLTGGDIYGMVHENTLVKHKMILPPKS
KGTVTYVAEPGNYTVDEVVLETEFDGERSKYTMLQVWPVRQARPVSEKLPANHPLLTGQRVLDSLFPCVQGGTTAIPGAFG
CGKTVISQSLSKYSNSDVIVYVGCGERGNEMSEVLRDFPELTVEIGGETESIMKRTALVANTSNMPVAAREASIYTGITLSEYFR
DMGYNVSMMADSTSRWAEALREISGRLAEMPADSGYPAYLGARLASFYERAGRVKCLGNPDREGSVSIVGAVSPPGGDFS
DPVTSATLGIVQVFWGLDKKLAQRKHFPSINWLISYSKYMRALDDFYDKNFPEFVSLRTKVKEILQEEEDLSEIVQLVGKASLA
EADKITLEIAKLLKEDFLQQNSYSSYDRFCPFYKTVGMLKNMIGLYDMSRHAVETTAQSENKITWNVIRDSMSNILYQLSSMK
FKDPVKDGEAKIKADFDQLYEDIQQAFRNLED

>Dvir_XP_050506367.1

MSKVRIGDEEKEGQYGYVHAVSGPVVTAEKMSGSAMYELVRVGYYELVGEIIRLEGDMATIQVYEETSGVTVGDPVLRTGKP
LSVELGPGIMGSIFDGIQRPLKDICDATDSIYIPKGINVPSLSRTAKWDFNPINIKLGSHLTGGDIYGLVHENTLVKHKMILPPRA
KGTVTYIAEPGNYTVDEVVLETEFDGDRTKYTMLQVWPVRQARPVSEKLPANHPLLTGQRVLDALFPCVQGGTTAIPGAFG
CGKTVISQSLSKYSNSDVIIYVGCGERGNEMSEVLRDFPELTVEIDGHTESIMKRTALVANTSNMPVAAREASIYTGITLSEYFR
DMGYNVSMMADSTSRWAEALREISGRLAEMPADSGYPAYLGARLASFYERAGRVKCLGNPDREGSVSIVGAVSPPGGDFS
DPVTTATLGIVQVFWGLDKKLAQRKHFPSVDWLGSYSKYLRALDDFYDKNFQEFIPLRTKVKEILQEEDDLAEIVQLVGKASL
AETDKITLEIARLLKEDFLQQNSYSSYDRFCPFYKTVGMLRNMIGLYDMARHAVESTAQSENKITWNVIRDSMSGILYQLSSM
KFKDPVKDGEAKIKADFDQLYEDIQQAFRNLED

>Tcas_XP_976188.1

MTSLPKMGDEERENKFGYVFAVSGPVVTAEKMSGAAMYELVRVGYSELVGEIIRLEGEMATIQVYEETSGVTVGDPVLRTGK
PLSVELGPGIMGSIFDGIQRPLKDINELTQSIYIPKGVNVPSLSRTTKWEFAPLNIKLGSHLTGGDIYGIVHENTLVKHKMLLPPK
AKGTVTYVADPGNYTVDEVVLETEFDGERTKYTMLQVWPVRQPRPVSEKLPANHPLLTGQRVLDSLFPCVQGGTTAIPGAF
GCGKTVISQSLSKYSNSDVIIYVGCGERGNEMSEVLRDFPELTVEIEGQTESIMKRTALVANTSNMPVAAREASIYTGITLSEYF
RDMGYNVSMMADSTSRWAEALREISGRLAEMPADSGYPAYLGARLASFYERAGRVKCLGNPDREGSVSIVGAVSPPGGDF
SDPVTSATLGIVQVFWGLDKKLAQRKHFPSINWLISYSKYTRALDDFYDKNFQEFVALRTKVKEILQEEEDLSEIVQLVGKASL
AETDKITLEIAKLLKEDFLQQNSYSSYDRFCPFYKTVGMLKNMIGLYDMSRHAVESTAQSENKITWTVIRDSMSNILYQLSSM
KFKDPVKDGEAKIKADFDQLYEDIQQAFRNLED

>Agra_XP_050303281.1

MSKLPKIGDEEREGEYGYVHMVSGPVVTAEKMSGSAMYELVRVGYNELVGEIIRLEGDMATIQVYEETSGVTVGDPVLRTG
KPLSVELGPGIMGSIFDGIQRPLKDINDLTQSIYIPKGVNVPCLSRTAQWEFNPVHIKMGSHLTGGDIYGMVHENTLVKHKMI
LPPKAKGTVTYIAEAGNYTVDDVVLETEFDGERTKYTMLQVWPVRQPRPVSEKLPANHPLLTGQRVLDSLFPCVQGGTTAIP
GAFGCGKTVISQALSKYSNSDVIIYVGCGERGNEMSEVLRDFPELTVEIDGATESIMKRTALVANTSNMPVAAREASIYTGITL
SEYFRDMGYNVSMMADSTSRWAEALREISGRLAEMPADSGYPAYLGARLASFYERAGRVKCLGNPDREGSVSIVGAVSPPG
GDFSDPVTSATLGIVQVFWGLDKKLAQRKHFPSVNWLISYSKYMRALDDFYDKNFPEFVALRTKVKEILQEEEDLSEIVQLVG
KASLAEGDKITLEVAKLLKEDFLQQNSYSAYDRFCPFYKTVGMLKNMIGLYDMARHAVESTAQSENKITWAVIRDSMSNILYQ
LSSMKFKDPVKDGEAKIKADYDQLYEDIQQAFRNLED

>Msex_XP_030028570.1

MASKGGLKTIANEENEERFGYVFAVSGPVVTAEKMSGSAMYELVRVGYNELVGEIIRLEGDMATIQVYEETSGVTVGDPVLR
TGKPLSVELGPGILGSIFDGIQRPLKDINELTQSIYIPKGVNVPSLAREVDWEFNPLNVKVGSHITGGDLYGIVHENTLVKHKML
MPPRAKGTVTYIAPAGNYKVTDVVLETEFDGEKAQYTMLQVWPVRQPRPVTEKLPANHPLLTGQRVLDSLFPCVQGGTTAI
PGAFGCGKTVISQALSKYSNSDVIIYVGCGERGNEMSEVLRDFPELTVEIEGVTESIMKRTALVANTSNMPVAAREASIYTGITL
SEYFRDMGYNVSMMADSTSRWAEALREISGRLAEMPADSGYPAYLGARLASFYERAGRVKCLGNPDREGSVSIVGAVSPPG
GDFSDPVTAATLGIVQVFWGLDKKLAQRKHFPSINWLISYSKYMRALDDFYEKNYPEFVPLRTKVKEILQEEEDLSEIVQLVGK
ASLAETDKITLEVAKLLKDDFLQQNSYSSYDRFCPFYKTVGMLKNIISFYDMSRHAVESTAQSDNKVTWNVIRDAMGNVLYQ
LSSMKFKDPVKDGEAKIKADFDQLLEDMSAAFRNLED

>Tabs_KAJ2937492.1

MSQKMKTVAGEENEERFGYVFAVSGPVVTAEKMSGSAMYELVRVGYNELVGEIIRLEGDMATIQVYEETSGVTVGDPVLRT
GKPLSVELGPGILGSIFDGIQRPLKDINELTQSIYIPKGVNVPCLARETAWEFNPLNVKVGSHITGGDLYGIVHENSLVKHKML
MQPRAKGTVTYIAPNGNYKVTDVVLETEFDGEKQSYTMLQVWPVRQPRPCTEKLPANHPLLCGQRVLDSLFPCVQGGTTA
IPGAFGCGKTVISQSLSKYSNSDVIVYVGCGERGNEMSEVLRDFPELTVEIDGVTESIMKRTALVANTSNMPVAAREASIYTGI
TLSEYFRDMGYNVSMMADSTSRWAEALREISGRLAEMPADSGYPAYLGARLASFYERAGRVKCLGNPDREGSVSIVGAVSP
PGGDFSDPVTAATLGIVQVFWGLDKKLAQRKHFPSINWLISYSKYMRALDDFYDKNYPEFVPLRTKVKEILQEEEDLSEIVQLV
GKASLAETDKITLEVAKLLKDDFLQQNSYSAYDRFCPFYKTVGMLRNIIAFYDMSRHAVESTAQSDNKVTWNVIRDAMGPTL
YALSSMKFKDPVKEGEAKIKADYDQLLEDMSAAFRNLED

>Hcun_UHA57771.1

MASDYQTIANEENEEKFGYVFAVSGPVVTAEKMSGSAMYELVRVGYNELVGEIIRLEGDMATIQVYEETSGVTVGDPVLRTG
KPLSVELGPGILGSIFDGIQRPLKDINELTQSIYIPKGVNVPSLARDVSWEFNPLEVKVGSHITGGDLYGIVHENTLVKHKMIVP
PKAKGTVTYIAPSGNYRCTDVVLETEFDGEKSSYTMLQVWPVRQPRPVAEKMPANHPLLTGQRVLDSLFPCVQGGTTAIPG
AFGCGKTVISQALSKYSNSDVIIYVGCGERGNEMSEVLRDFPELTVEIEGVTESIMKRTALVANTSNMPVAAREASIYTGITLSE
YFRDMGYHVSMMADSTSRWAEALREISGRLAEMPADSGYPAYLGARLASFYERAGRVKCLGNPDREGSVSIVGAVSPPGG
DFSDPVTAATLGIVQVFWGLDKKLAQRKHFPSINWLISYSKYMRALDDFYDKNYPEFVPLRTKVKEILQEEEDLSEIVQLVGKA
SLAETDKITLEVAKLLKDDFLQQNSYSAYDRFCPFYKTCGMLKNIIAFYDMSRHAVESTAQSDNKVTWNVIRDAMGNVLYTL
SSMKFKDPVKEGEAKIKADFDQLLEDMSAAFRNLED

>Sgre_XP_049841688.1

MDLIKSFQMTSTLIKTSDEDRESKFGFVFAVSGPVVTAERMAGSAMYELVRVGYYELVGEIIRLEGDMATIQVYEDTSGVTVG
DPVLRTGKPLSVELGPGIMGSIFDGIQRPLKDINELSNSIYIPKGVNVPALSRTAQWDFSPVSVKIGSHITGGDLYGLVHENTLV
KHKLLLPPRAKGTVTYIAEPGNYTVDDIVLETEFDGERSKFTMLQVWPVRQPRPVTEKLPANYPLLTGQRVLDSLFPCVQGG
TTAIPGAFGCGKTVISQSLSKYSNSDVIIYVGCGERGNEMSEVLRDFPELSVEIDGVTESIMKRTALVANTSNMPVAAREASIY
TGITLSEYFRDMGYNVSMMADSTSRWAEALREISGRLAEMPADSGYPAYLGARLASFYERAGRVKCLGNPDREGSVSIVGA
VSPPGGDFSDPVTTATLGIVQVFWGLDKKLAQRKHFPSINWLISYSKYMRALDDFYDKNFPEFVPLRTKVKEILQEEEDLSEIV
QLVGKASLAETDKITLEVAKLLKDDFLQQNSYSPYDRFCPFYKTVGMLKNMIAFYDMSRHAVESTAQSENKITWNVIRDSM
GNILYQLSSMKFKDPVKDGEAKIKADFEQLHEDIQQSFRNLED

>Btab_XP_018897790.1

MSNLAKIRDEERESKFGYVFAVSGPVVTAEKMSGSAMYELVRVGYFELVGEIIRLEGDMATIQVYEETSGVTVGDPVLRTGKP
LSVELGPGILGSIFDGIQRPLKDINEINQSIYIPKGVNIPALSKSHAWEFNPLNIKIGSHITGGDLYGIVFENTLVKHKLMLPPKAK
GTVTYIAPPGNYKVDDIVLETEFDGEQSKYTMLQIWPVRQPRPVTEKLPANHPLLTGQRVLDSLFPCVLGGTTAIPGAFGCG
KTVISQSLSKYSNSDVIVYVGCGERGNEMSEVLRDFPELTVEIDGVTESIMKRTALVANTSNMPVAAREASIYTGITLSEYFRD
MGYNVSMMADSTSRWAEALREISGRLAEMPADSGYPAYLGARLASFYERAGRVKCLGNPDREGSVSIVGAVSPPGGDFSD
PVTSATLGIVQVFWGLDKKLAQRKHFPSINWLISYSKYTRALDDFYDKNFPEFVALRSKVKEILQEEEDLSEIVQLVGKASLAES
DKITLEVAKLLKDDFLQQNSYSPYDRFCPFYKTVGMLRNMIAFYDMSRHAVESTAQSENKISWSVIKDSMGNILYQLSSMKF
KDPVKDGEQKIKADFDQLHEDIQQAFRNLED

dsRNase1 aa sequences

>Dmel_NP_649076.1

MKCIRFSLLVVGLLAAPAAWARVPCPEVELPPVVEDDGIFERIAVAPPQPVGRAGACSVTIRGGLPSPEPVYLKTDSEDFYPFS
DVGVMEFESGGSLQLWCPSGFNTHSENLLTASCVSGTTFSVGGSNFEFKDLYCKSWPGFKAVKSGATCNGGIVIRVGFEITSS
RFAEQMQICFNEEEEVTRYTRHKLEPGSNYYETGVARITFQTAGFFDGKNVDKLYTQATQLETINNELGGDAEKYFDSSSNVY
LARGHLGAKADFDYAPEQRATFLFINAAPQWQTFNAGNWARVEDGLRAWVSKNKLNVNCYTGVYGVTTLPNKDGVETPL
YLAKDDNNNGLIPVPKLYFRVVIDPSSHRGIVFVGVNNPHLTEEQIKRDYVICDDVSDQVTYINWKTTDIKAGWSYACEVADF
LKTVKHLPALTAKGGLLV

>Ccap_XP_004530585.1

MKLTTTLLLLVTGSCCLLQGCTAGVIAVPEVVIDGLKIDEEKPEESVFERNLYVEPISGAEDDYVEPEDELLPPAPVVDDKPKGR
ATACKVTIRGGLPTPQPVYLKSDSAEFYPYDSTGVMVVESGEKLDLWCPGKFTSLDKTLVSASCVSGTNFKVDGTTYTLKELTC
KSWPSFVAEKTGSSCNGGVEVRVGFKVSSSRFVEQYKVCFDEDEEVTRYVHHDLNPGSNYYQTGVDRLTFQTGGFFDGKN

VDKLYTQATQLTTINEQLGGDASKYFDSSKNVYLARGHLAAKADFDYGTEQRATFLFINAAPQWQVFNAGNWARIEDGVR
AKVSKAQWYVDCWTGVYGVTTLANANGVQTPLYLAYDSNNNGLIPVPKLYFRVVIERTTKKGIVFIGVNNPHLTLAEIKKDYI
LCTDVADKVDYVNWKPTDITAGYSYACEVDDFKKKVSHLPDLPSVTGLLV

>Btry_XP_039968826.1

MKLTSTLLLLVAGSFCLFHGCTAGVVAVPEDVVDDWKADDHMSEQINTLFERNFYVEPIKEDASQAIMPAEVEEDYVEPEPIS
EVEDELVQPEVDVNGKIEGRASECKVTIRGGLPTPQPLYLKSGSEEIYPYDTKGVMVVDAGSTLEMWCPGKFTTLDTTLVTAT
CVSGTNFRVDGTTYAFKELTCKAWPTFVAEKTGASCNGGIMVRVGFKISSSRFAQQYEVCFNEGEEVTRYVHHDLNPGANY
YQTGVDRITFQTGGFFDGKSVDKLYTQATQLATINEHLGGDASKYFDSAKNVYLARGHMAAKADFDYGLEQRATFLFINAAP
QWQVFNAGNWARIEDGVRAKVSSAGWYVDCYTGVYGVTTLPNSDGVQTPLYLAYDSNNNGLIPVPKLYFRVVIEKTSKKGI
VFIGVNNPHLTLDEIKKDYILCTDIADQVNYVSWKRTDLTAGYSYACEVSDFRSKVTNLPNLSATGGLLL

>Zcuc_XP_011186396.1

MKLSSTLLLLVAGSFCLFHGCAAGVVAIPQLVIDEAGEQPPEAIVPISNEEDKLEGLETLFERNLYVEPIQSGDDEYVKPVPISVG
EAEEDYVEPEPIDEVEDELVQPPEVDVNAKPEGRASECKVTIRGGLPNPQPVYLKSGSNEFYPYDASGVMVVDSGRTLELWC
PGKFHSIDKTLVTASCVSGSNFRVDGTTYSFKELSCKSWPGFVAEKTGASCNGGIMVRVGFKISSSRFVEQYQVCFNEGEEVT
RYVHHDLNPGSNYYQTGVERLTFQTGGFFGGKNVDKLYTQVTQKETINNHLGGDANKYFDNNKNIYLARGHMAAKADFD
YGTEQRATFLFINAAPQWQVFNAGNWARIEDGVRAKVSKSKWYVDCYTGVYGVATLPNNRGVETPLYLSYDGNNNGQIPV
PKLYFRVVIERSSKKGIVFIGVNNPHLSMDDIKRNYVICDDVADRVNYINWKRTDITAGYSYACEVSDFRRKVTHLPNLSAPGG
LLL

>Alud_XP_053954646.1

MKLTTSLLLLVAGLSCLFEGCAAGVSVIPQNAIDDLKYRDQSAEPIAPVNVDEEIVPIFERSINVEPINGGQEDYVEPAPIAEVE
AASVESKSEVEDESVKPESDANEILVGRASECKVAIRGGLSSPQPVYIKKGSTEFYPYDDSGVMVVDSGSSLEMWCPGKFSSI
SQTLVTATCVSGTNFKIDGTTYAFKELACKAWPTFTAEKTGASCNGGIMVRVGYKVSSSRFIEQYTVCFDEVEEVTRYVHHDL
NPGSNYYETGVARITFTTGGFFDGKAVNTLYTQVTQQETISAALGMDSSKYFDYSTSLYLARGHLAAKADFDYAAQQRASFLF
INAAPQWQTFNAGNWASVEDSLRSKVTKSKWTVDCYTGVYGVTTLPNSEGVQTPLYLAYDNNNNGLIPVPKLYFRVVIERS
TNKGIVFIGVNNPYLTLDEIKKDYIICTDVADQVDYITWKRTDITAGYSYACTVSDFMSKVSHLPSLSASGGLLL

>Ldec_APF31792.1

MFLFSVTLVLTLQFSFLSGAPSATECEIDPFSANAPLLIENGKSSIIYPQYGERTIRFPAGHVVELSCPQREVLTNGESSGSIVVAT
CVSGGRFKMNGQTFNWEKIVCSGVPVSTSRITNGKCGKNGQSAEIGFNLDNSRFLTTMEICFDTVKQIALYSHFEMSSTISIS
SSDTPRPQFHEDSGFYHLNGEKVNNLYVRKGQRKTINGLLGLSTDSVKYIQNGVLYLARGHLTARADFVYAAEENSTFKYINA
APQWQSINAANWKQVEMDTRNYANAHKVGLQVWTGTYGVASLPHEKTGKDIELYLYANSDYSAIPVPALYWKIVYNPVNK
RGIVLIGVNNPFLKEEQMKKYIICRDVSDSVSWLKWKKSNIAHGYSYACTVPDFRKVVTYAPSLTVSGLLDT

>Dvir_XP_050501052.1

MVVMCNCVVLVGVTSILLYLVALIHADCQIDPFDGKAPLVLTAKDSSIVYPNSGETTLDFRNGEIVTFACSGNNIFLSGLMHQT
TVEGRCLANSQFDVFGKRYSWTDIACSSNPRATIRKTNSHCARDATMVVVGYDLGNGNFASIIDICFNTSSQIALYSRYDITSS
IQSNDETFSRPAFFEDSNLYNIKGRVDTYYKQNRQRTTINNLLGLSPTSSKYISSGDLFLSRGHLAAKTDFLYGFQQDATFRYIN

VAPQWQSFNGGNWNRVERSCRNYADRRKANLQIWTGTYGIATLPHEFTQKPTELYLYVNGRTKALPVPALYWKIVYNPSNF
RCVVLIGLNNPFEDNVSRYIICRDISNSLNWISWQKNDHKKGYSYACTCNDFKSRVDYAPSLKVSGILN

>Tcas_XP_970494.2

MLLPALFLLLQVSLGDFFILRAPDCNIQISNLDPEPIVVDGTYTFLYAAPDASSVLVKSGETIIISCPGGEITVGSTSFNSTVSATCV
SNSDFSVGSATINFNQIVCSWNPFHTARYTGKSCEKQGKEIEVGFVINENFAREITICFDNANLNTLYSSYEITKSIGHHESGVS
RPFFIEDDFYNLDVKVNSLYVRGGQRTTINSLLGLPADSTKYIQDGNDFYLARGHFAAKADFVYAPQQTATFHYVNVAPQW
QSFNGYNWNQVESDVRDYAEKNGIDLKMYTGTYGVTTLPHEETGEETPLYLYIGSNGIQGIAVPELYWKVAYNPETQLGVAL
LGINNPYQKDINKSIICEDVSAKINWLHWNASDTKAGYSYACEVDAFRKRVTYLPDFVVKGLLL

>Agra_XP_050311286.1

MLLLLTVLLSSVATSFATPVQDLRLDNTASVDLTAVACEINPFATTPAPLLVRNSNFVYPSGAGRSTITFAAGETIDFACPGGRLV
LDGASTTLQVATASCVSGTRFIVNNARYLWNQVRCSANPVSSSRYTGRTCEAGGREAEIGFAITTSRFARTMLICFDAARQTTI
YSYYDLIPAINQQITGTPRPSWTQGSGMFTLNNVNTLYTTATQRVTINGLLGLSSTSTTVIQNNNYFLSRGHLTARSDFFYAAQ
QNSTFHFLNALPQWQTFNGFNWDQAEKDVQDYAERNNAQLQVWTGGFNMTTLPHSRTGVETELYLSVSGSQRLLPVPD
LFWKVVYNPTTKRGVALIGVNNPYKTANQINRFCDDRSDLLTWLNWRKNDQARGISWACTVPAFRRIVNSFPNVEISGLLF

>Msex_XP_030023618.2

MKRAVVLFSALVLAVYSLPFSLPEPGQLALLLSEDDFEDYLDTWLEIEGTKQANILRSSNRNGCSIRVNGDLGQPQPVYLQGN
NYLIPSGNTGIIHLNTGEQVYLACTGSNRLLRHPNISASTATGTATCVNNSLVSGAGWLNGHGEFGQITCSSHSEHNAEGTPQ
RCYNNNLVIRVGFIVHGVFYPLYWSCFDQYRLEVLYVWYEQNPQNAAHQTSVDRPNWLAGSFFPGVSVNNMYTQAQQK
TTIAKLVGTDLANKYITSTQYLARGHLAAKTDFIYATGQRATFYFINSAPQWQPFNAGNWNWLEQNLRKRIGEAGYNTIVYT
GTFGVTQLRAQNNQLVDIYLYYDVNNNPQIPVPLYYYKVVYDSSRRIGTAFVGVNNPYYTESEMRTLQFCTDRCRNNSAFN
WIGWQPDRIDLGYSFCCTINDFRRVVPHLPSFTVNGLLS

>Tabs_KAJ2950598.1

MARLLFIIAALAAAAAALPAAMERPPMPEPGELALLLNEDEFEDYLDQWLEWEQQRNSNVSTADVARNNGCRFRVNGDL
GQPQPLYLHNNNFIVPTGNTGVINLNTGQAVTIACAGNGRTIKHPRISSTVAVANAVCVNNNLVSGSGWLKGNGAFGGLTC
SSHSHHDAEYTSERCFNNNRVIRVGFQVQGQFYTKFRSCFDPNRLEVLYTWYEQNPQNAVNQAGVDRPSWLAGSFFTGV
GINQRYTQAEQKRMIASYVGQTLANKYVTSSQFLARGHLTAKTDMIFATGQRSTFYFINAAPQWQPFNAGNWNWLEQNL
RRRIGEAGYHTTIYTGTFGVTQLRDQNNRLVDIYLHRDSNNNPQLPVPQYFYKVVYDASRRLGTAFISINNPHYTESEVRALQ
FCTDRCRNNSAFKWIGWQPDRIDIGYSFCCTVADFRRVVPHLPNFQVNGLLS

>Hcun_UVJ48452.1

MGPLVVFVAFLAVAAGFELPHPSRFAKVFNEDQFEDYLDFWLAQEEPKWANASSSLRHSEISSRSSGCTLNINGDLGQPQP
VYIRSGTYLRPTGNSGQIHLNTGDQVIIGCPGRTLRHPNIASTTLIVGTATCVSGNLVSGSGWLNGNGAFGELTCSSHSFHEA
QATNNRCWGNNLVIRIGFIVNNVFHTLYHSCFNEQRMEVIYVEYEQNPENGIHQTGVDRPSFLAGSFFPGIAVNTRYTQVE
QKKTVAEYVGQTLADFYITSHQFLARGHLAAKTDFVFATGQRASFYFINAAPQWQPFNAGNWNTLEQNLRARIAAANYRT
TVYTGTFGVSQLRDQSNRLVDIYLVRDKNQIPVPLYFYKVVYDASRRQGTAYVSINNPYYTEAEARSLTFCTDLCRNNSAFSWL
RWQPDRIDIGYSFCCTVEDFRRTVPHLPAFTTTGLLT

>Sgre_XP_049863887.1

MTRPADAPGRAGGCSVDINDESAFPDPQPLFLLPGGSADAKGFWEPEVSSELLTLQEGDQLNLACTGSGNVLTVLGVAEAT
ATCVSGTTFSVDGRSYALSDLKCSSLPDSSQRDTQQTCGASAGVYPLLQIGFELSTGFVKLIDICFDDTRLTALYAQETIVAGIGG
YQSGFPRPNWMQGDFFGDIDVNTAYTQNKQRETISEILGSSELGDQYVTSGDYYLSRGHLSAKADWVFGSQQTATFWFLN
AAPQWQTFNGANWETVESNVRYYASKKGADLTVYTGTYGVATLPNVDGVETELYLVADTKQIPVPKLYWKVVHDAANDAG
VALIGINNPYVSDPGDDYYLCPDVCSKLSWVTWESNDQKKGFSYCCEVSEFQKVVSVLPEISVSSLPTL

>Btab_CAH0381107.1

MIISLAPILVVVFSLHGYVGAVCKVSLNNNLPTKEPLYLTHKGSKLDFVYPKSAKGASKRDKGSFELSENEELVFACPGKANKLA
RTTENAADSHCVSGTRFSLGAKKTSNIEQIECTSSVKATLELDSGKKCAKTGTQMKIGFEVERQLLPLMWVCHDIKAADTIFV
EHDIPATIGGARIFKARPDFEDGPSHLYEGINVKNVYTQKYQRGLFNQLLGKGQGEKFIKGTYYLARGHLAPDGDFLYGSWQ
WSTYFYVNTAPQWQIINAGHWLALERYLRKFAEQTGEDLHIMTGIVGVLSFESDSGENVEIYLQPDEQKIRVPAAFFKVIRSE
VSDRAIVAVCSNNPFEKVPTLCQDIAAEHRWPTSWHDHAKGHIYFCEVNDFLSNASGLPIRRNSGILRGIGK

dsRNase2 aa sequences (only Diptera)

>Dmel_NP_648610.1

MWSNRCSIFILALLVASGAVKVSGRLPRILPESLAEGDEGVTVRGDCEFKVNGDLNDPAPLFSRHNSYEIIVPDPTDTVRLVNG
ELLDMFCPGVGFAAPFVNRWQVTATCLQNKYFLVDDLIYPFANFSCTAWPIFTALRSGKDCNGGTDLVQVGFEVEDGGFLQ
SYELCHDAEAEATRYVHHVLYPSSYDYQHGVARPNFLELDFYGGRDVNTKYTQVQQNITISNILGLDASPYFNFSDDRILSRG
HMIAKTDQIFGAAQHTTFLFINVAPQWQTFNGGNWEKVETSVRKFVADRNLTTDCYTGTWGVSTLPDVDGIERELYLDFD
ENNNGLIPVPKIYFRVVIDRVTREGIVLIGINNPYLTLEQIQKDYILCQDIGHQLSWLTWYKEDLHEGYSYACSVEDFIEVVKDLP
LEDLHTNGILGLDDVTTVEPSTTESSTTTEEPSSTTSTEIPSTTDTTPSSTSVSSTTEEPSSSTASPTTELPTSTVISTTEPPTSTVTS
TTKPPTSTVASTTEQPTSTVTSTTEPPTSTVTPTTEPPTSTVTSTTEPSSSTTLEPTSTTSSSTTSQPTLSTEESTSTSPTSTSTSSPP
VTTANPIANPCRFSTNGDLKDPAPLLTPQGALSWLLPDESGLYTVEEGSSIDLHCTGALVSPFNRYTALSARCVGDQLYEAEGQ
LVNIRGFVCQSWPTYAAVRSGQSCAGGTDVVHIGFDVAAGFLSHVELCYDQQEQISRYARYELTPANVAYQKGVAQPGYLR
GDFYSGEDVNVLYDQSHQLDAFSTALGLDASQYFDSTKDLYLTRGQLAARLDFVHSSAQRATHFYVNAVPQWRSINIGNWL
AVESSLKQFVADEALNVSVHAGSYDISTLPNSQGVQTPLYLNAETKQLPVAKILYRIVIDQESRKGVVLIVVNNPHITLTETLEDY
VICEDVGAQIDWLDWDKANLQKGYSYACSVEDFIEVVKDLPTDQLQTTGILGLTDQILDNEVCSFKVNGDLKDPAPLFVVRS
ELGHARYLEPNQEGVVQLKHGEALEFHCINSFSGPFSGYTFVNSTCWQQQSILAMGSLYQLQDFVCTSWPTYTARRSGRPC
NGGTDLLEVGFQLSSSSSDDFLQTYDVCHDELSEVTRYVHHVLYPGSDQYQRSVSRPSFIPGDFYGGKDVNTLYTQVQQNIT
VSEILGMDASPYFNTTGNVYLARGHLSAKTDFVFGAAQKASFFFVNAAPQWQTFNGGNWERIEDSVRKFVADENITVDCY
TGTWGVSTLPDVNGIGRELYLDFDENNNGLIPVPKLYFRVIIDRESRNGIVLLGVNNPHATIEQIEEEYIICQDIGAQLSWVSW
TKEDLKKGYSYACTVEDFTAVVKDLPLQDLFVDGVLGV

>Ccap_XP_004530581.1

MSLVKYSVPLLVTLLCSLVSIEAGVAQSKNREYYGPSTPAITVTYPTPPSLIITQGPETNTTVEPTFSSSTLTTDGPVFSTTTTPNT
TTSQAIPPPPTKTTRPTQPPHTPPTPPPPTQPPTPPTNGGVVVRGDCAFDVNGDLNDPAPIFMPHNQLEWLVPNPDGVVE
LGNGALVDMYCNKSFVAPFTNRTKVTAQCLQKQYFLVDGVIYPFSNFSCTDWPAFTARRTGRVCNGGTDLLEVGFELEAGFL
QTMDICHDEVNEVTRYVHHVLNPSSAGYQHGVSRPRFITGDFYNGKNVDNLYTKVKQNQTFSEILGMDATPYFNDTIDVYL
ARGHMAAKVDFIFGAPQKATFYFVNVAPQWQMFNGRNWERIEDGVRRFASDQALTLDCYTGTWGISKLPDVNGVPQEL

YLAFDENNNGLIPVPMLYFRVVIDRASRKGIVLLGVNNPHATLEEIQRDYVICKDVGRRITWISWDKENLANGYSYACAVDDF
TSVVKDVPLENLHTTGLLGVEELKIENIPIWPEL

>Btry_XP_039967124.1

MYSGGKAVKLVLVLACLLAVVEAGILHSKETVVKSLLANTENDGPRTPYPTLSTANTYPTPPYINVTDGTTDVTDETTDVTTD
GPFSSTTPPPEIGGGVVVRGDCAFDVNGDLNDPAPIFTPQNQLEWLVPNPAGVVELSNGAYIDMYCDKSFIAPFSNLTKVTA
QCLQKQYFLVDGVIHPFSDFSCTSWPAYTARRTGRPCNGGTDLVEVGFVLTSGFLQIMDVCHDEVNEVTRYVHHKLNPSSA
GYQHGVTRPSFITGDFYAGKNVNNLYTKVEQNNTISKILGMDASPFFNDTIDVYLARGHMAAKVDFIFGAPQKATFFFVNA
APQWQMFNGRNWERVEDSVRRYASDQALDLDCYTGIWGVSTLPDVNGVQRELYLDFDENNNGLIPVPKIYFRVVIDRKSR
NGIVLIGVNNPHVTLEEIKKDYVICKDVGNRIKWVSWDKENLMNGYSYACAVDDFISVVKDLPLDDLYTSGLLGVEELTIENIP
S

>Zcuc_XP_011186392.2

MYTVVNAVKLALALVCTLAVVEAGILYSKEPVVKTFILDDGPPTAYPTLSTNFTYPTPPSLNVTDGPTTATTDGPYNSTTPPPPE
ISGGVVVRGDCAFDVNGGLNDPAPIFTPHNQLEWLVPNPAGVVELSNGAYIDMYCSKSFMAPFSNRTKVTAQCLQKQYFLV
DGLIYPFSNFSCTDWPGYTARRTGRTCNGGTDLLEVGFELSAGFLQTMDICHDEVNEVTRYVHHKLNPSSAGYQHGVSRPR
FITGDFYAGKNVDNLYTKKQQNQTFSNILGMDASPFFNDTIDVYLARGHMAAKVDFIFGAPQKATFYFVNAAPQWQMFN
GRNWERVEDGVRRYASTQALDLDCYTGIWGISTLPDVNGVQRELYLAFDENNNGLIPVPKIYFRVVIDRKTRKGIVLIGVNNP
HASLEEIQRDYVICKDVGKRINWISWDKENLKNGYSYACGVEDFISVVKDLPLDELYTTGLLGVEEIKIENIPL

>Alud_XP_053956955.1

MELAKRTVALLFALICLLAAVQAGLLQSKARSRREESVTIATLGTTDTYSTPPTESPINGTTEGPITSSIPPNTTTPALPSTTDAPP
PENGTGVVVHGDCAFDVNGDLNDPAPIFMLSNRLEWIQPNSAGVVVLSNGAYVDMYCSKSFMAPFTNRTKVTAQCLQKQ
YFLVDGLIYPFSNFSCTSWPAHTARRTGRFCNGGTDLLEVGFELEVGFLQTMDVCHDEVNEVTRYVHHVLNPASNSYQHGV
TRPSFIPGDFYAGKNVDNLYTKVKQNETISKILGMDASPYFNDSINVYMARGHMAAKADFIFGAAHKATFYFVNAAPQWQ
TFNGGNWERIEDSIRRYATDQALTLDCYTGIWGVSTLPDVNGVEQELYLAFDENNNGLIPVPKLYFRVVIDRESRKGIVLLGV
NNPHLTVEEIQSDYVICKDVGKRIDWIGWDKENLMKGYSYACAVDDFTQVVKDLPLEDLYTTGLLGVEELKIENIPIWEEH
